# Immunoreactivity of Lupine and Soybean Allergens in Foods as Affected by Thermal Processing

**DOI:** 10.3390/foods9030254

**Published:** 2020-02-27

**Authors:** Caterina Villa, Mónica B. M. V. Moura, Joana Costa, Isabel Mafra

**Affiliations:** REQUIMTE-LAQV, Faculdade de Farmácia, Universidade do Porto, Rua de Jorge Viterbo Ferreira, 228, 4050-313 Porto, Portugal; caterinavilla@hotmail.com (C.V.); mbvmoura@gmail.com (M.B.M.V.M.)

**Keywords:** *Lupinus albus*, *Glycine max*, proteins, food processing, meat products, bakery products

## Abstract

Lupine and soybean are important technological aids for the food industry. However, they are also capable of inducing severe allergic reactions in food-sensitized/allergic individuals. In this context, this work intended to study the combined effects of thermal processing and food matrix on the immunoreactivity of lupine and soybean proteins used as ingredients in bakery and meat products, respectively. For this purpose, the effects of baking, mild oven cooking, and autoclaving on the protein profiles were evaluated, using model mixtures simulating the production of lupine-containing breads and soybean-containing cooked hams/sausages, by native- and sodium dodecyl sulfate-polyacrylamide gel electrophoresis (SDS-PAGE), and immunoblotting using specific antibodies. The results showed that lupine gamma-conglutin immunoreactivity was slightly decreased in wheat flour mixtures compared to rice, but it was more pronounced in baked products. In meat mixtures, substantial protein fragmentation was noted after autoclaving, with decreased immunoreactivity of soybean trypsin inhibitor. The analysis of 22 commercial products enabled the identification of lupine gamma-conglutin in four bakery samples and soybean trypsin-inhibitor in five sausages, and further differentiated autoclaved from other milder thermally treated products. Generally, the immunoreactivity of target proteins was reduced by all the tested thermal treatments, though at a higher extent after autoclaving, being slightly altered by the food matrix.

## 1. Introduction

Legumes, belonging to the Fabaceae family, are consumed worldwide due to their high contents of proteins and essential elements, including vitamins and lipids. However, they play an important role in the scenario of food allergy, with an increased sensitization to legumes among populations from Mediterranean and Asian countries, as well as from western countries, in the last years [1]. Immunoglobulin E (IgE)-binding proteins have been identified in the majority of legumes, being responsible for causing mild skin reactions to life-threatening anaphylactic shocks in sensitized individuals after their ingestion or inhalation. Lupine and soybean are important legumes capable of inducing severe allergic reactions. Lupine has been ranked ninth in the category of “risk allergens” [2] while soybean is one of the eight groups of foods accountable for about 90% of food-allergic reactions [1,3]. Among sensitized children, 6% were diagnosed with soybean allergy while an overall prevalence of 2.1% was found in a study involving several European countries, the USA, and Australia [1,4,5]. Regarding lupine allergy, the prevalence varies, depending on dietary habits and geographical differences [1,6].

The addition of lupine and soybean protein materials as food ingredients has been increasing due to their important nutritional and technological characteristics, such as emulsifier properties, gelling capability, texture improvement, and water-binding property [7]. These materials are classified as protein isolates (PI, >90% protein content), protein concentrates (PC, 65%–90% protein content), or protein flours (PF, 50%–65% protein content), being frequently added to several food products by food manufacturers [8]. Lupine protein materials can be isolated from four *Lupinus* species, namely *L. albus* (white lupine), *L. angustifolius* (blue or narrow-leafed lupine), *L. luteus* (yellow lupine), and *L. mutabilis* (pink or Andean lupine) [9], while soybean protein materials are normally extracted from *Glycine maxima* species. Lupine flour is a common ingredient used in the production of bakery and pastry products, whereas soybean protein isolate (SPI) and soybean protein concentrate (SPC) are often found in meat products, such as cooked hams and sausages [7]. To protect sensitized consumers, the European regulations about food labelling included soybean and lupine as allergenic foods that must be emphasized in the list of ingredients of pre-packaged foods [10]. The enforcement of labelling legislation helps allergic consumers adopting an elimination diet avoid the risk of having adverse immunological reactions caused by the inadvertent ingestion of the offending food.

Most of the food products containing lupine or soybean are submitted to thermal treatments (baking, boiling, roasting, autoclaving, microwave heating, etc.) during their industrial production or cooking at home. It is reported that heat treatment of food proteins may produce different modifications, such as hydrolysis of peptide bonds, denaturation, aggregation by disulfide and non-covalent bonds, and reactions with other molecules present in the food matrix, namely lipids, sugars, or carbohydrates [11]. Such modifications might affect the integrity of epitopes recognized by specific IgE, influencing protein allergenicity, by either enhancing (exposure of epitopes or generation of new ones) or reducing it (loss of epitopes), possibly leading to an altered capacity to elicit an allergic reaction [3]. Some studies have been developed in order to find strategies to reduce lupine [12,13,14,15,16] and soybean [17,18,19,20,21,22] allergenicity by the application of thermal processing technologies. The literature shows that heat treatments can affect lupine and soybean allergenicity differently, depending on a wide range of factors that include the duration of the process, the intensity, and the presence of a food matrix. Besides processing, the effect of the food matrix is also of major importance, in particular with regard to the interactions of allergens with other food components that might alter their properties [23].

In this context, the aim of this work was to study the combined effects of thermal processing and the food matrix on the immunoreactivity of lupine and soybean proteins used as ingredients in bakery and meat products, respectively. For this purpose, the effects of baking, oven cooking, and autoclaving were evaluated after their application to model mixtures simulating the production of lupine-containing bread and cooked hams and sausages containing soybean PI (SPI) and soybean PC (SPC), respectively, using immunochemical assays. Commercial samples that stated the presence of lupine or soybean proteins in their list of ingredients or were suspected of containing them were also tested.

## 2. Materials and Methods

### 2.1. Sampling

Flour of *L. luteus* was provided by the company Germisem (Coimbra, Portugal) and *L. albus* (Biosagesse, France) was acquired at a local market while the seeds of *L. mutabilis* (accession no. 90-0581D) and *L. angustifolius* (accession no. PI180708) were provided by the US National Plant Germplasm System (NPGS) through the National Genetic Resources Program of the US department of Agriculture (NGRP-USDA) (WA, USA). The soybean materials (SPI and SPC) used in this study were provided by FORMULAB (Maia, Portugal). For antibody specificity testing, several food species (*n* = 10) reported to be cross-reactive with lupine and soybean were evaluated, namely wheat, tree nuts (hazelnut and walnut), legumes (peanut, fava bean, bean, chickpea, pea, and lentil), and milk. Twenty-two commercial foods labelled as containing lupine (cookies, cakes, wafers, and bakery products) or soybean (cooked hams and sausages) were acquired at different Portuguese retail markets and are described in Table 1.

### 2.2. Model Mixtures and Sample Preparation

Several model mixtures spiked with known amounts of lupine or soybean materials were prepared. Lupine flour (*L. albus*), SPI, and SPC were used as lupine and soybean materials, respectively. The exact protein contents of each material were obtained by the Kjeldahl method [24,25]. Regarding lupine reference mixtures, two independent sets of binary model mixtures containing 10.0%, 1.0%, 0.1%, 0.01%, and 0.001% (*w/w*) of lupine flour in rice or in wheat flours were prepared [25]. The first mixture containing 10% of lupine protein was prepared by adding the required amount of lupine flour to 200 g of rice or wheat flours. The following mixtures were prepared by successive stepwise additions of the respective matrix flour.

For the preparation of model breads, 180 g of water, 4.5 g of salt, 6 g of baker’s yeast, and 3 g of bread improver were added to 300 g of each binary mixture of lupine in wheat flour. Model wheat breads containing 10%–0.001% (*n* = 5, *w/w*) of lupine flour were prepared as a reference. Two additional model wheat breads containing 2.5% and 0.25% of lupine flour were also prepared to simulate commercial samples (Table 1). Doughs were cooked in a bread machine Moulinex OW6101 (Ecully, France) for 3 h with a maximum temperature of 180 °C for 25 min. After cooling, the breads were cut in the middle to remove slices, which were ground in the laboratory knife mill (Grindomix GM200, Retsch, Haan, Germany).

For the preparation of soybean reference mixtures, two recipes were followed, simulating the production of cooked hams and sausages (Frankfurt type). The preparation of raw hams included minced raw pork meat (1.0 kg), salt (8.0 g), and sugar (4.0 g) while raw sausages were prepared with minced raw pork meat (500 g), salt (20 g), crushed ice (250 g), and pork lard (375 g). The first mixture containing 10% of SPI was prepared by adding 13.7 g of soybean material to 106.3 g of raw ham while the one containing 10% of SPC was prepared by adding 18.5 g of soybean material to 101.5 g of raw sausage, both prepared using a laboratory knife mill (Grindomix GM200, Retsch, Haan, Germany). To facilitate homogenization, 10 mL of sterile phosphate-buffered saline solution (136 mM NaCl, 1.4 mM KH_2_PO_4_, 8.09 mM Na_2_HPO_4_·12H_2_O, and 2.6 mM KCl, pH 7.2) were added to each mixture. The following mixtures were prepared by successive additions to obtain 10-fold dilutions in the concentration range of 10%-0.001%, similarly to lupine mixtures. Each mixture was divided into 2 portions: The first portions were immediately stored at −20 °C (raw ham and sausage mixtures); the second portions containing SPI were submitted to oven cooking at 68 °C for 5 h to simulate the industrial processing of cooked hams while the second portions containing SPC were autoclaved for 15 min, at 121 °C (autoclaved sausages), to mimic the industrial processing of sausages.

All commercial samples were minced and homogenized with the laboratory knife mill (Grindomix GM200, Retsch, Haan, Germany). All materials and different blender containers were previously treated with a decontamination solution in order to avoid contaminations. All samples were immediately stored at −20 °C until further analysis.

### 2.3. Protein Extraction and Quantification

Prior to protein extraction, two buffers (PBS 0.2 M, pH 7.4 and Tris-HCl 100 mM, pH 8.0) were tested for their suitability to extract good quality protein from all model mixtures and commercial samples. Better protein extracts were obtained with Tris-HCl buffer (100 mM, pH 8.0), being the elected buffer for extracting proteins in this work. Briefly, 150 mg of sample were weighted and 1.5 mL of Tris-HCl buffer were added, followed by an incubation at 60 °C for 2 h, with stirring at 950 rpm and frequent vortexing to increase the protein yield. After incubation, the mixtures were centrifuged twice at room temperature (9000 g, 30 min). Between centrifugations, the supernatant was collected, and the pellet discarded in order to provide clear supernatants. After extraction, the protein concentration was assessed by UV spectrophotometry on a Synergy HT multi-mode microplate reader (BioTek Instruments, Inc., Winooski, VT, USA), using a Take3 micro-volume plate accessory and the protein280 protocol in the Gen5 data analysis software version 2.01 (BioTek Instruments, Inc., Winooski, VT, USA).

### 2.4. SDS-PAGE and Native-PAGE Analysis

Sodium dodecyl sulfate-polyacrylamide gel electrophoresis (SDS-PAGE) (5–12%) or native-PAGE (5–12%) gels in discontinuous system were homemade, following the protocols described in the Mini-PROTEAN^®^ Tetra Cell Instruction Manual [26]. All model mixtures and samples were run at 150 V in denaturing and native conditions, using a Mini-PROTEAN^®^ Tetra System (Bio-Rad Laboratories, Inc., Hercules, CA, USA), with 1× Tris/Glycine/SDS (Bio-Rad Laboratories, Inc., Hercules, CA, USA) or 1× Tris/Glycine (Bio-Rad Laboratories, Inc., Hercules, CA, USA) electrophoresis buffer, respectively. Electrophoresis under reducing conditions was carried out by adding 2× Laemmli Sample Buffer (Bio-Rad Laboratories, Inc., Hercules, CA, USA) containing 50 mM β-mercaptoethanol to each sample in a 1:1 ratio, followed by denaturation for 5 min at 95 °C. For native conditions, Native Sample Buffer (Bio-Rad Laboratories, Inc., Hercules, CA, USA) was mixed in a 1:1 ratio with each sample, followed by direct application on native-PAGE gels. Protein quantity loaded into gels ranged from 1 to 15 µg of protein per lane. The proteins were visualized by staining the gels with Coomassie Brilliant Blue G-250 solution or blotted into a nitrocellulose membrane (for further immunoblot analysis). A gel image was collected using a white tray and processed with Image Lab 5.2.1 software (Gel DocTM EZ Imager, Bio-Rad Laboratories, Inc., Hercules, CA, USA). Precision Plus Protein™ Dual Color Standards (10–250 kDa, Bio-Rad Laboratories, Inc., Hercules, CA, USA) was used as a protein molecular weight reference.

### 2.5. Immunoblotting Analysis

After electrophoresis, the gels were blotted into nitrocellulose membranes 0.2 µm transfer pack (Bio-Rad Laboratories, Inc., Hercules, CA, USA), using the Trans-Blot^®^ Turbo™ Transfer System (Bio-Rad, Laboratories, Inc., Hercules, CA, USA) with an automatic turbo protocol (2.5 A, up to 25 V, 10 min). To verify the efficiency of the transfer, membranes were colored with Ponceau S 0.1% solution for 10 min. After acquiring an image of Western blot membranes, they were washed with TBST 1× (pH 7.4, 10 mM Tris, 50 mM NaCl, 0.1% Tween 20) at least 3 times, 10 min each, until the red coloration disappeared. Then, the membranes were blocked with TBST 1× containing 2% of gelatin from cold-water fish skin (Sigma-Aldrich, St Louis, MO, USA) for 1 h at room temperature, with constant and gentle agitation. After blocking, the membranes were washed 3 times for 10 min with TBST 1×. The membranes were incubated overnight at 4 °C with a primary antibody specific to soybean (rabbit anti-trypsin inhibitor antibody, Abcam, Netherlands) or to lupine (rabbit anti-conglutin gamma globulin antibody, Agrisera, Sweden) diluted 1/40,000 or 1/50,000 in incubation buffer (TBST 1× with 2% fish gelatin), respectively. The anti-rabbit IgG peroxidase antibody produced in goat (Sigma-Aldrich, St Louis, MO, USA), diluted 1/40,000 in incubation buffer, was applied to the membranes for 1 h at room temperature. Between each incubation with antibodies and at the end, the membranes were washed for 10 min (3×) with TBST 1×. The immunoreactive proteins were revealed with Clarity™ Western ECL (Bio-Rad Laboratories, Hercules, CA, USA) for a few minutes and chemiluminescence was acquired on a ChemiDoc system (the membrane was normally instantly revealed).

## 3. Results and Discussion

In this study, different model mixtures were prepared containing lupine or soybean protein materials. In the case of lupine, two different matrix flours (wheat or rice) were used to prepare model mixtures in order to evaluate the matrix effect. A third set of model mixtures was prepared using the lupine flour in the wheat dough, which was baked to simulate the production of bread (to assess the baking effect). The mixtures for the cooked hams and sausages (both raw and processed) with the addition of SPI and SPC, respectively, were also used. The protein profiles of the raw and processed model mixtures were compared and the immunoreactivity of lupine or soybean proteins was assessed by the use of specific polyclonal antibodies targeting the lupine gamma-conglutin or the soybean trypsin-inhibitor, which were critically selected from the available commercial antibodies. Both proteins, the gamma-conglutin (lupine) or the trypsin-inhibitor (soybean), are classified as allergens by the ALLERGOME database [27]. Similarly, commercial samples containing lupine and/or soybean proteins were also analyzed.

### 3.1. Antibody Specificity

Despite their high specificity, antibodies are raised in biological systems (animals), and as such, they can present some unintended reactivity with none target proteins, meaning that it is very important to determine their experimental specificity. Patients allergic to lupine or soybean often suffer from adverse immunological responses upon contact with proteins from other food sources, particularly legumes [28]. Peanut, lentils, beans, peas, and chickpeas are all legumes of the same botanical family (Fabaceae or Leguminosae), which explains their protein homology and the presence of highly similar epitopes with subsequent IgE recognition [29]. Therefore, in order to evaluate the specificity of both primary commercial antibodies (anti-conglutin gamma-globulin and anti-soybean trypsin inhibitor) and to avoid any false positive results, all the referred species were tested. Moreover, wheat and rice were also included in this evaluation, since they were used as food matrices in model mixtures, as well as three additional species of lupine commonly used as food ingredients (*L. luteus*, *L. mutabilis*, and *L. angustifolius*).

**Figure 1:**
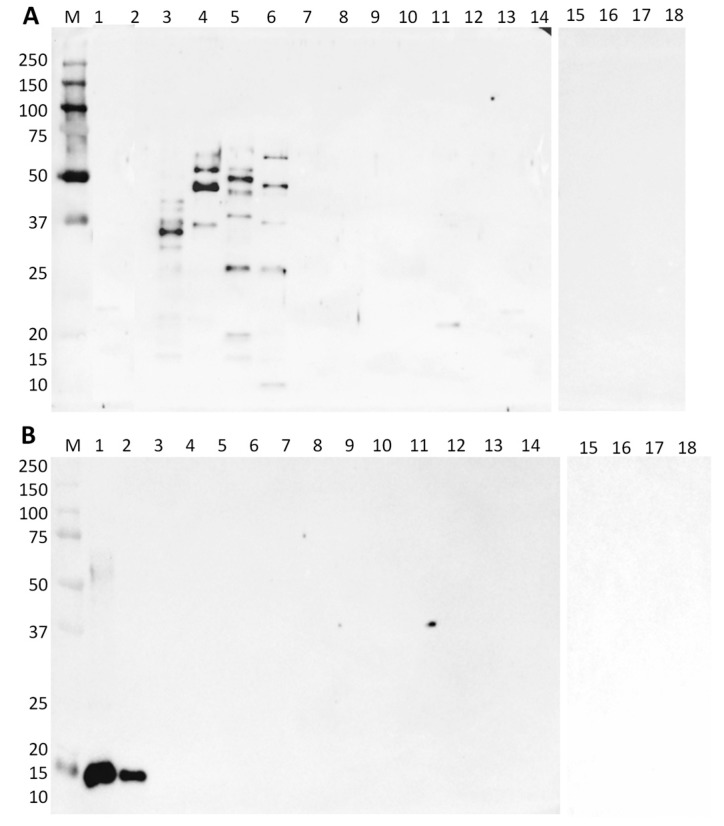
Immunoblot membranes using anti-gamma-conglutin polyclonal antibody (**A**) and anti-trypsin inhibitor polyclonal antibody (**B**) for the specificity test with different plant species. Legend: lane 1, soybean protein isolate (SPI); lane 2, soybean protein concentrate (SPC); lane 3, *L. albus*; lane 4, *L. luteus*; lane 5, *L. mutabilis*; lane 6, *L. angustifolius*; lane 7, rice; lane 8, wheat; lane 9, maize; lane 10, milk protein concentrate; lane 11, peanut; lane 12, walnut; lane 13, hazelnut; lane 14, fava bean; lane 15, chickpeas; lane 16, bean; lane 17, lentil; lane 18, pea; lane M, Precision Plus Protein Dual Color Standard 10–250 kDa (Bio-Rad Laboratories, Inc., Hercules, CA, USA).

Regarding the specificity of the commercial anti-lupine antibody, for the same protein quantity of each species (2.5 μg), all four species of lupine presented different immunoreactive bands (Figure 1A, lanes 3–6). Gamma-conglutin was already identified as an allergenic protein of lupine [15,30], although it can show unspecific binding properties [31]. Lupine gamma-conglutin is a lectin-like glycoprotein, with a high affinity for galactose, which can bind N-glycosylated proteins, for example, Fc fragments of IgE, but without eliciting clinical symptoms and thus giving, in some cases, false positive results regarding allergy diagnosis [32]. Different patterns of immunoreactive bands among the four species of lupine might be explained by the different degree of protein glycosylation and the presence of distinct subunits according to the target species. Some bands can be identified as corresponding to the allergenic gamma-like large and small subunits of the protein, namely at 29 and 17 kDa, respectively (Figure 1A, lanes 3, 5, and 6), as already reported by Magni et al. [30]. Two bands also appeared at 43 kDa, which might coincide with the unreduced gamma-conglutin of *L. albus* (Figure 1A, lane 3). Moreover, bands with approximately 50 kDa can be observed in *L. luteus*, *L. mutabilis*, and *L. angustifolius*, already identified as the gamma-conglutin precursor [31]. A strong band at approximately 37 kDa in *L. albus* seems to be the most reactive, also observed by Holden et al. [15] in lupine flour using sera from lupine-allergic patients. So far, allergenic gamma-conglutins have been identified in different *Lupinus* species, namely in *L. albus* (Lup a gamma-conglutin) and *L. angustifolius* (Lup an gamma-conglutin) [27], being in good agreement with the immunorecognition of gamma-conglutins in all the tested species. None of the other tested plant species or milk reacted with anti-lupine antibody, except for peanut, where a band of weak intensity was observed at approximately 20 kDa (Figure 1A, lane 11). This anti-gamma conglutin antibody recognized the target allergenic protein in all tested lupine species, thus allowing their unequivocal identification.

In the case of anti-soybean antibody, only soybean materials (isolate and concentrate) presented a band at 21 kDa, corresponding to the allergenic soybean trypsin inhibitor [27], thus confirming the specificity of this antibody for unequivocal soybean identification (Figure 1B, lanes 1 and 2).

### 3.2. Effect of the Food Matrix and Heat Processing

#### 3.2.1. Lupine Proteins

For the evaluation of the effect of the food matrix on lupine proteins, model mixtures containing known amounts of lupine flour in wheat or rice flours were prepared. To simulate the production of bread and evaluate the effect of the heat treatment, dough containing wheat model mixtures were submitted to a baking process for 3 h with a maximum temperature of 180 °C during 25 min. The protein profiles (10 µg loaded in each lane) (Supplementary Material, Figure S1) have different patterns according to the food matrix used in the preparation of model mixtures. SDS-PAGE results show that the protein profile of rice flour presents visible bands only at 75, 45, 37, and 17 kDa (Supplementary Material, Figure S1A, lane 8), while wheat flour exhibits complex band patterns (Supplementary Material, Figure S1A,B, lane 14). In native conditions, both matrices present bands with high molecular weights Supplementary Material, Figure S1C,D, lanes 8 and 14). Moreover, the protein profiles of model mixtures are clearly affected by the baking process since most of the protein bands from the breads appear to be almost fully degraded (Supplementary Material, Figure S1B,D).

**Figure 2:**
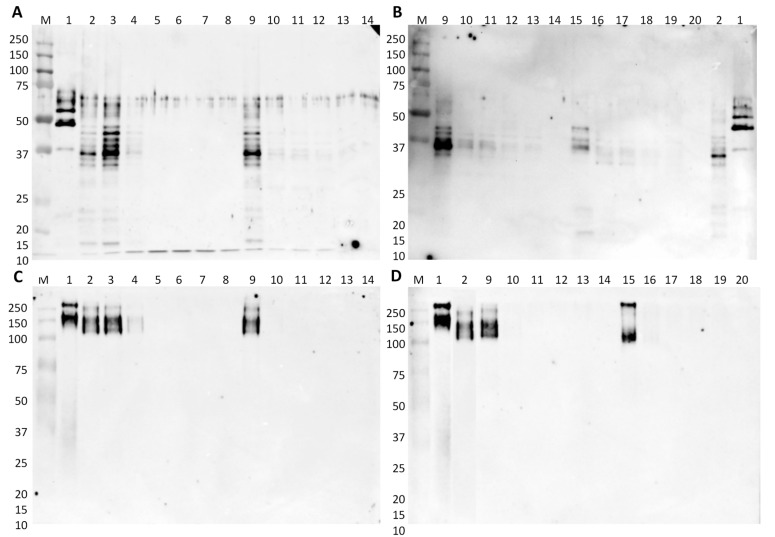
Immunoblot membranes for lupine testing using anti-gamma-conglutin polyclonal antibody in denaturing (**A**,**B**) and native conditions (**C**,**D**) comparing rice and wheat flours model mixtures (**A**,**C**) and wheat flour with model breads (**B**,**D**). Legend: lane 1, *L. luteus*; lane 2, *L. albus*; lanes 3–7, 10%, 1%, 0.1%, 0.01%, and 0.001% of *L. albus* in rice flour; lane 8, rice flour (0% lupine flour); lanes 9–13, 10%, 1%, 0.1%, 0.01%, and 0.001% of *L. albus* in wheat flour; lane 14, wheat flour (0% lupine flour); lanes 15–19, 10%, 1%, 0.1%, 0.01%, and 0.001% of *L. albus* in bread; lane 20, wheat bread (0% lupine flour); lane M, Precision Plus Protein Dual Color Standard 10–250 kDa.

The immunoreactivity of the lupine material in the model mixtures was then investigated by immunoblotting with the specific anti-conglutin antibody. Membranes contained 0.5 or 2.5 μg of the total protein for raw or processed model mixtures, respectively, are shown in Figure 2. As previously highlighted, the immunoreactive bands of *L. albus* in denaturing conditions are between 30 and 50 kDa, with the most intense at approximately 37 and 43 kDa, with the latter one probably corresponding to the unreduced form of gamma-conglutin (Figure 2A,B, lane 2). When analyzed in native conditions, a single smeared band at 200 kDa can be observed, corresponding to the proteins in their native state (Figure 2C,D, lane 2). Comparing the use of rice and wheat flours as matrices in denaturing conditions, for the same protein quantity in each lane (0.5 μg), it is possible to observe a stronger immunoreactivity in model mixtures prepared with rice than with wheat flour. However, in lupine/wheat flour mixtures, the immunoreactive bands are still visible at 0.1% of lupine (Figure 2A,B, lane 11) while in lupine/rice flour mixtures the signal is visible until 1% of lupine (Figure 2A, lane 4). In native conditions, the band corresponding to lupine gamma-conglutin is visible until 1% or 10% in rice or wheat flours, respectively (Figure 2C, lanes 4 and 9), which might be related with the conformational structure of native protein. Regarding the effect of processing on the immunoreactivity of lupine gamma-conglutin, the results obtained in denaturing conditions show a clear negative effect of heat treatment, mainly at higher lupine proportions, with a reduction of the intensity of target bands, but still with visible signals until 0.1% of lupine in both raw and processed model mixtures (Figure 2B, lanes 11 and 17). In native conditions, the same negative effect is observed, but with immunoreactive bands only in the 10% lupine mixtures (Figure 2D, lanes 9 and 15).

In general, a reduction of lupine immunoreactivity was observed after the baking treatment at 180 °C, with significant effects on the integrity and structure of lupine gamma-conglutin. As already reported by Álvarez-Álvarez et al. [12], autoclaving at 138 °C for at least 20 min induced a reduction in the overall allergenicity of lupine seeds while boiling, microwave, and extrusion cooking did not produce any modification. Holden et al. [15] used *L. albus* seeds in a tofu-like product (Lopino), which was prepared with soaked seeds that were blended, whose filtrate was boiled and pressed. The subsequent IgE-binding capacity of the resultant lupine proteins, assessed using sera from lupine-allergic patients, was decreased in heat-treated lupine. In opposition to this, Álvarez-Álvarez et al. [12] verified an important reduction of the IgE-binding capacity of lupine only after prolonged autoclaving. This fact can be explained by the use of a real food matrix that might modulate the changes caused by the processing, thereby affecting the allergenicity of lupine proteins because the induction of an IgE response depends on their intrinsic properties, as well as the matrix in which they are administered [15,33].

#### 3.2.2. Soybean Proteins

The preparation of cooked hams and sausages involves industrial processes that apply distinct heat treatments. In the case of cooked hams, the industrial process includes a slow oven cooking (5 h) using moderate temperature (68 °C) while for sausages the thermal treatment is more severe with the application of high temperatures (121 °C) and pressure for a short period of time (15 min). Model mixtures of pork meat containing known amounts of soybean materials (SPI and SPC) were prepared, simulating both treatments of mild oven cooking and autoclaving. The protein profiles of raw and processed mixtures were then compared by PAGE in denaturing and native conditions. The effect of oven cooking and autoclaving on the protein profiles (15 µg of protein in each lane) of all model mixtures is clearly shown in Figure S2 (Supplementary Material). In denaturing conditions, most protein bands from processed soybean and pork meat appear to be almost fully degraded, although this effect is more drastic in autoclaved sausages (Supplementary Material, Figure S2B, lanes 21–26) than in cooked hams (Supplementary Material, Figure S2A, lanes 8–13). It is also important to highlight that there are several bands corresponding to high molecular weight proteins (150–250 kDa) in cooked hams, preserving the same profile as in raw hams (Supplementary Material, Figure S2A), suggesting that oven cooking only partially affects the integrity of some proteins. When analyzing the protein profile of processed model mixtures in native conditions, only few bands are visible at the molecular weight around 75 kDa, which might result from the aggregation of smaller proteins (Supplementary Material, Figure S2C,D, lanes 8–13). Like in denaturing conditions, the autoclaving process (Supplementary Material, Figure S2D, lanes 21–26) seems to be more drastic in altering the conformation and size of the proteins.

**Figure 3:**
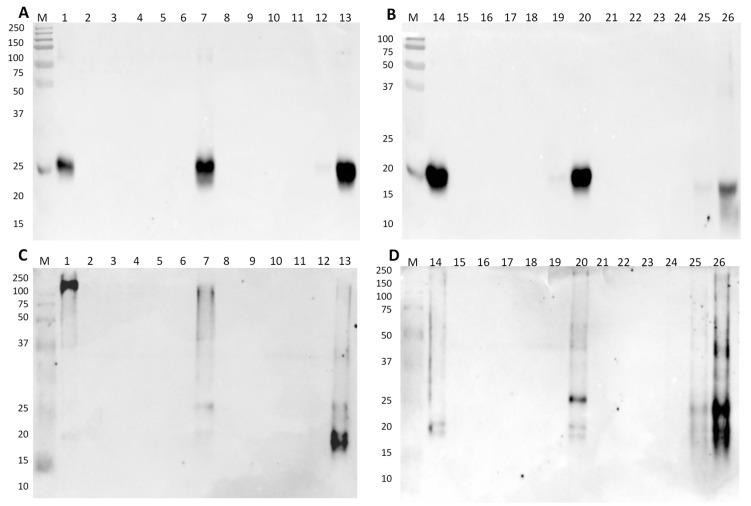
Immunoblot membranes for soybean testing using anti-trypsin inhibitor polyclonal antibody in denaturing (**A**,**B**) and native conditions (**C**,**D**) with model mixtures simulating raw and cooked hams (**A** and **C**) and raw and autoclaved sausages (**B** and **D**). Legend: lane 1, SPI; lane 2, raw pork ham (0% SPI); lanes 3–7, 0.001%, 0.01%, 0.1%, 1%, and 10% of SPI in raw pork ham; lane 8, cooked-pork ham (0% SPI); lanes 9–13, 0.001%, 0.01%, 0.1%, 1%, and 10% of SPI in cooked pork ham; lane 14, SPC; lane 15, raw pork sausage (0% SPC); lanes 16–20, 0.001%, 0.01%, 0.1%, 1%, and 10% of SPC in raw pork sausage; lane 21, autoclaved pork sausage 0% SPC; lanes 22–26, 0.001%, 0.01%, 0.1%, 1%, and 10% of SPC in autoclaved pork sausage; lane M, Precision Plus Protein Dual Color Standard 10–250 kDa.

The immunoblotting results of model hams and sausages with soybean materials, using membranes containing 1.5 or 15 μg of total protein for raw or processed model mixtures, respectively, incubated with a polyclonal antibody against soybean trypsin-inhibitor, are presented in Figure 3. In denaturing conditions, intense bands of 21 kDa identifying the soybean trypsin inhibitor (target protein) are recognized in SPI (Figure 3A, lane 1), and in the model mixtures of 10% of soybean material in raw (Figure 3A, lane 7) and cooked ham (Figure 3A, lane 13). A faint band is also identified in the 1% SPI mixture in cooked ham run in denaturing conditions (Figure 3A, lane 12). In the model sausages (Figure 3B), the results are similar to raw hams, with the identification of strong immunoreactivity in the SPC and 10% SPC mixtures of both raw and autoclaved sausages (Figure 3B, lanes 14, 20, and 26). A very weak signal is identified in the autoclaved 1% SPC mixture (Figure 3B, lane 25), which is due to the 10-fold higher concentration of this sample compared to its raw counterpart. The results in native conditions are in good agreement with the denaturing conditions, although the pattern of the soybean trypsin inhibitor seems slightly different, probably due to the protein total charge (Figure 3C,D). The bands at 17–18 kDa of the soybean trypsin inhibitor in cooked hams and autoclaved sausages (Figure 3C,D, lane 26) might result from partial degradation during processing. There are also some very weak bands at higher molecular weights (25 and 40 kDa) in processed mixtures (Figure 3C,D, lanes 13 and 26), a fact that might be justified by the formation of reactive aggregates during heat treatments. In native conditions, trypsin inhibitor may undergo conformational alterations at mild temperatures, leading to a molten globule structure (structure that preserves partial spatial conformation, native-like secondary structure) [34]. The formation of such globular structures during thermal processing can expose some hidden epitopes or create new allergenic determinants, increasing protein immunoreactivity.

### 3.3. Analysis of Commercial Samples

#### 3.3.1. Lupine-Containing Products

A set of commercial samples containing lupine as an ingredient, such as bakery and pastry products, were analyzed by SDS-PAGE in denaturing conditions, native-PAGE, and immunoblotting to assess the molecular structure and immunoreactivity of lupine proteins. The protein profiles of the analyzed food samples are presented in Figure S3 (Supplementary Material), together with the four main *Lupinus* species and relevant flours frequently used as ingredients of those foods for comparative purposes. Using the same amount of proteins (10 µg per lane), the highly processed samples like biscuits, cookies, and breads exhibit fragmented protein patterns, including mostly faint/smeared bands because of being submitted to harsh processing conditions (Supplementary Material, Figure S3A,B, lanes a–e, h–k). Contrarily, the flour samples for making bread present more complex protein patterns (samples #g, #l, and #m). Cookies and breads showed generally similar patterns due to the presence of wheat flour proteins, with more intense bands at 50 and 70 kDa (samples #c, #f, #h, #j, and #k). In native conditions, most of the samples present smeared bands or no bands, pointing out to protein fragmentation owing to severe processing, while the few bands at high molecular weight might be aggregates formed during baking (Supplementary Material, Figure S3C,D).

**Figure 4:**
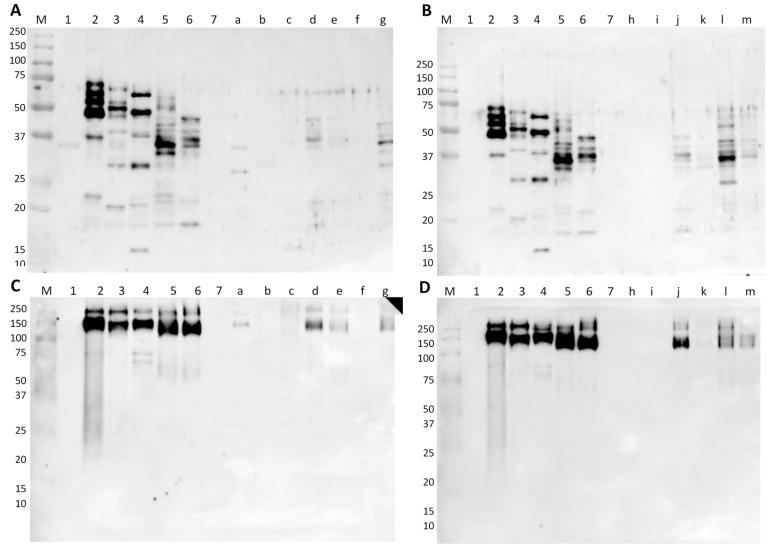
Immunoblot membranes for lupine testing using anti-gamma-conglutin polyclonal antibody in denaturing (**A**,**B**) and native conditions (**C**,**D**) with commercial and in-house-made samples. Legend: lane 1, SPI; lane 2, *L. luteus*; lane 3, *L. mutabilis*; lane 4, *L. angustifolius*; lane 5, *L. albus*; lane 6, 10% of *L. albus* in bread; lane 7, wheat bread (0% lupine flour); lane a, cereal bread; lane b, cookies; lane c, lupine biscuits; lane d, Pan Carré; lane e, “crostini”; lane f, lupine cookies with lemon flavor; lane g, flour for bread; lane h, cookies; lane i, chocolate wafers; lane j, model bread containing 2.5% of lupine flour; model bread containing 0.25% of lupine flour; lane l, flour mixture containing 2.5% of lupine in wheat; lane m, flour mixture containing 2.0% of lupine in rice; lane M, Precision Plus Protein Dual Color Standard 10–250 kDa.

From the immunoblotting results (5 µg of protein per lane), it was possible to identify immunoreactive bands of gamma-conglutin in four commercial samples, namely in a cereal bread, Pan Carré, “crostini”, and flour for bread, both in native and denaturing conditions (Figure 4A,C, samples #a, #d, #e, and #g). The two bands, at approximately 37 and 27 kDa in sample #a, seem to be different from the bands of samples #d, #e, and #g, which exhibit patterns close to *L. albus*. These bands could correspond to *L. mutabilis* or *L. angustifolius*, highlighting the potential use of these two lupine species in processed foods. Sample #a stated “may contain traces of lupine” in its label while the other three mentioned lupine protein, which is in good agreement with the obtained results. Samples that declared lupine flour as an ingredient, but without exhibiting any reactivity, were probably submitted to treatments that were more aggressive or have undetectable lupine proteins by the antibody. The model wheat breads containing 2.5% and 0.25% of lupine, as well as the flour mixtures with lupine, presented immunoreactive bands corresponding to the protein pattern of *L. albus*, with the raw wheat flour mixture being the most reactive in denaturing conditions (Figure 4B, sample #l). Contrarily, in native conditions, the most reactive bands are observed in bread (Figure 3D, sample #j). In these conditions, the proteins might form aggregates with increased immunoreactivity, probably as result of the formation of new conformational epitopes [35].

#### 3.3.2. Soybean-Containing Products

Different commercial samples of cooked hams and sausages made of pork or poultry meats were analyzed by PAGE, both in denaturing and native conditions (Supplementary Material, Figure S4). In general, the protein profiles of cooked hams are clearer and with well-defined molecular weight bands (Supplementary Material, Figure S4A,C) than the ones of autoclaved sausages (Supplementary Material, Figure S4B,D), which might be due to the differences in the thermal treatment. In autoclaved sausages (canned), the high temperature in combination with high pressure might have contributed to form aggregated macromolecules with very high molecular weight that can explain the intense smeared bands at 150–250 kDa in the pork sausage samples (Supplementary Material, Figure S4B,D, lanes 13, h, I, and j). In the bottled poultry sausages (autoclaved), the intense smeared bands are also visible, though at lower molecular weights (Supplementary Material, Figure S4B,D, lanes k–l). The vacuum-packed sausages (Supplementary Material, Figure S4B,D, lanes n and o) have been subjected to a boiling treatment, much softer than autoclaving, presenting protein profiles that are close and consistent with the profiles of poultry cooked hams (Supplementary Material, Figure S4B,D, lanes e–g). Like in the case of autoclaved model mixtures, the protein profiles of commercial autoclaved samples in native conditions (Supplementary Material, Figure S4C,D) are smeared bands, denoting protein fragmentation. Generally, these results highlight that the protein structure was more significantly affected by the severe thermal processing of autoclaving (Supplementary Material, Figure S4D) than by soft cooking/boiling (Supplementary Material, Figure S4C).

**Figure 5:**
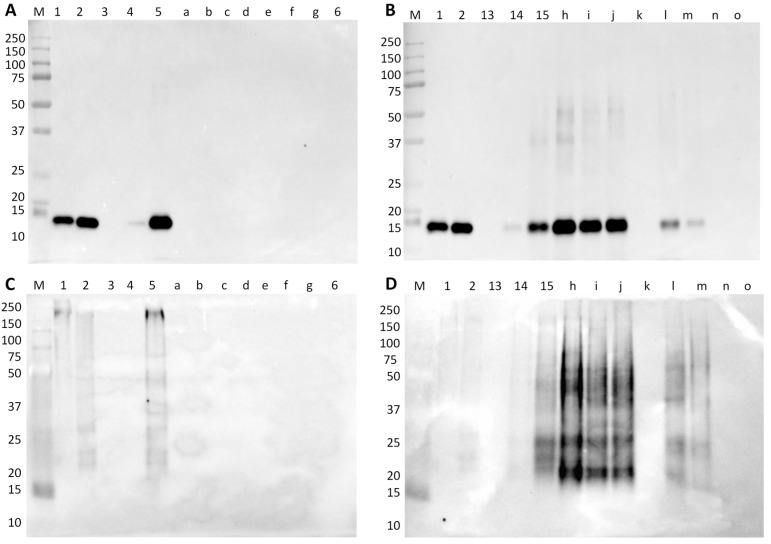
Immunoblot membranes for soybean testing using anti-trypsin inhibitor polyclonal antibody in denaturing (**A**,**B**) and native conditions (**C**,**D**) with hams (**A**,**C**) and sausages (**B**,**D**) commercial and in-house-made samples. Legend: lane 1, SPI; lane 2, SPC; lane 3, model pork cooked ham (0% of SPI) (negative control); lanes 4,5, pork cooked ham with 1% and 10% of SPI (positive control); lanes a–c, commercial pork cooked hams; lane d, model turkey cooked ham (0% of SPI); lane e, commercial turkey cooked ham; lane f, commercial chicken cooked ham; lane g, commercial turkey cooked ham; lane 6, milk protein concentrate; lane 13, model pork sausage (0% of SPC); lanes 14,15 model autoclaved pork sausage with 1% and 10% of SPC; lanes h–j, commercial pork sausages; lane k, model turkey sausage (0% of SPC); lane l, commercial turkey sausages; lane m, commercial chicken sausages; lane n, commercial turkey sausages; lane o, commercial turkey and chicken sausages; lane M, Precision Plus Protein Dual Color Standard 10–250 kDa.

The commercial samples of cooked hams and sausages were also tested by immunoblotting, targeting the soybean allergen trypsin inhibitor (Gly m TI). In both denaturing and native conditions, no immunoreactivity to soybean can be observed for all cooked-ham samples (pork and turkey/chicken) (Figure 5A,C). All these samples were labelled as “fiambre da perna extra”, which according to the Portuguese Standard [36] are products that cannot contain proteins from vegetable origin. Thus, these samples were not expected to contain soybean proteins (at least considering the qualitative result in this type of immunoassay). With respect to Regulation (EU) No 1169/2011 [10] that establishes the mandatory labelling of soybean and products thereof, among other potentially allergenic foods (including soybean), the statement of “may contain or contain soybean” (Table 1) suggests the practice of precautionary labelling since soybean was not detected. From the seven samples of commercial sausages, five of them presented strong immunoreactivity to soybean trypsin inhibitor protein (around 21 kDa) (Figure 5B). In the canned samples (#h, #i, and #j), the amount of soybean protein seems to be higher than 10%, considering the band intensities when compared with the respective model mixture of 10% SPC in autoclaved pork sausage (Figure 5B, lane 15). The two bottled samples, #l and #m (Figure 5B, lanes l and m), present bands with low intensities, though higher than the 1% SPC model mixture (Figure 5B, lane 14), while in vacuum-packed samples soybean was not detected. These findings are consistent with the estimated higher amounts of soybean material in the canned sausage samples than in bottled ones, suggesting that the lower cost canned products contain more soybean probably for cost reduction [37]. According to Portuguese Standards defined for the characteristics of Frankfurt-type [38] and raw sausages [39], a maximum addition of 5% of vegetable proteins is recommended. Therefore, to verify if sausage samples are according to that recommendation, a quantitative analysis should be performed to determine the amount of soybean protein [37]. Nevertheless, they are all in good agreement with Regulation (EU) No 1169/2011 [10], regarding the labelling of soybean ingredients (Table 1). The immunoblot results obtained in native conditions (Figure 5C,D) confirmed all data obtained in denaturing conditions (Figure 5A,B).

## 4. Conclusions

In summary, with this work, it was possible to evaluate the effect of food processing on the immunoreactivity of lupine and soybean proteins used as technological ingredients in food products. All the tested thermal treatments, namely baking, mild oven cooking, and autoclaving, were able to reduce the immunoreactivity of target proteins, although at greater extension in the case of autoclaved sausages compared to cooked hams. The food matrix was also proven to affect the immunoreactivity of allergenic proteins since lupine/rice flour mixtures presented more intense bands compared to bands of lupine in wheat, suggesting the interaction of lupine proteins with wheat molecules, which can decrease IgE binding. Therefore, the importance of the use of model mixtures to simulate, as much as possible, the processing and matrix of a real food was clearly demonstrated. More studies about the effect of processing in food allergens are needed, mainly towards the development of strategies able to reduce protein allergenicity.

## Figures and Tables

**Table 1:** List of commercial and model samples tested in this work.

Sample	Type of Product	Relevant Label Information
Lupine		
a	Cereal bread	May contain traces of lupine
b	Cookies	May contain traces of lupine
c	Lupine biscuits	Lupine flour
d	Pan Carré (bread)	Lupine protein
e	Crostini (mini toasts)	Lupine protein
f	Lupine cookies with lemon flavor	Lupine flour
g	Flour for bread	Lupine protein
h	Cookies	Sweet lupine flour
i	Chocolate wafers	Sweet lupine flour
j	Bread	Model bread (containing 2.5% of lupine flour)
k	Bread	Model bread (containing 0.25% of lupine flour)
l	Flour for bread	Flour mixture (containing 2.5% of lupine flour)
m	Rice flour	Flour mixture (containing 2.0% of lupine flour)
Soybean		
a	Pork cooked ham (Fiambre da perna extra)	Flavors (milk, gluten, soybean)
b	Pork cooked ham (Fiambre da perna extra)	Flavors (contains gluten and soybean)
c	Pork cooked ham (Fiambre da perna extra)	Flavors (contains soybean products)
d	Turkey ham	Model cooked ham
e	Turkey breast ham	Soybean protein (may contain traces of milk)
f	Chicken breast ham	May contain traces of milk protein and soybean
g	Turkey breast ham	Milk protein (may contain traces of soybean)
h	Pork sausages (canned)	Soybean protein
i	Pork sausages (canned)	Soybean protein
j	Pork sausages (canned)	Soybean protein
k	Turkey sausage	Model sausages
l	Turkey sausages (bottled)	Soybean protein (may contain traces of milk)
m	Chicken sausages (bottled)	Soybean protein (may contain traces of milk)
n	Turkey Frankfurt sausages (vacuum packed)	May contain traces of soy and milk protein
o	Turkey and chicken sausages (vacuum packed)	No information about soybean (Gluten and milk “free”)

## Supplementary Materials

The following are available online at https://www.mdpi.com/2304-8158/9/3/254/s1, SDS- and native-PAGE containing protein profiles of model mixtures prepared with soybean or lupine technological materials and of commercial foods are presented as in the supplementary material section (Figures S1–S4). Figure S1: SDS-PAGE run in denaturing (A, B) and PAGE native conditions (C, D) comparing model flour mixtures of lupine in rice and lupine in wheat flours (A, C) and model breads with lupine in wheat flour (B, D), Figure S2: SDS-PAGE gels in denaturing (A and B) and PAGE native (C and D) conditions comparing model mixtures simulating pork hams (A and C) and pork sausages (B and D), Figure S3: SDS-PAGE run in denaturing (A, B) and PAGE in native conditions (C, D), Figure S4: SDS-PAGE gels run in denaturing (A and B) and PAGE native (C and D) conditions with commercial samples of cooked-hams (A and C) and sausages (B and D).
