# Supplementary material for: Immunoreactivity of Lupine and Soybean Allergens in Foods as Affected by Thermal Processing
Source: Foods. 2020 Feb 27;9(3):254. doi: 10.3390/foods9030254 (PMC7142587; doi:10.3390/foods9030254)
Supplement: Supplementary file 1 [file foods-09-00254-s001.pdf]

Supplementary material

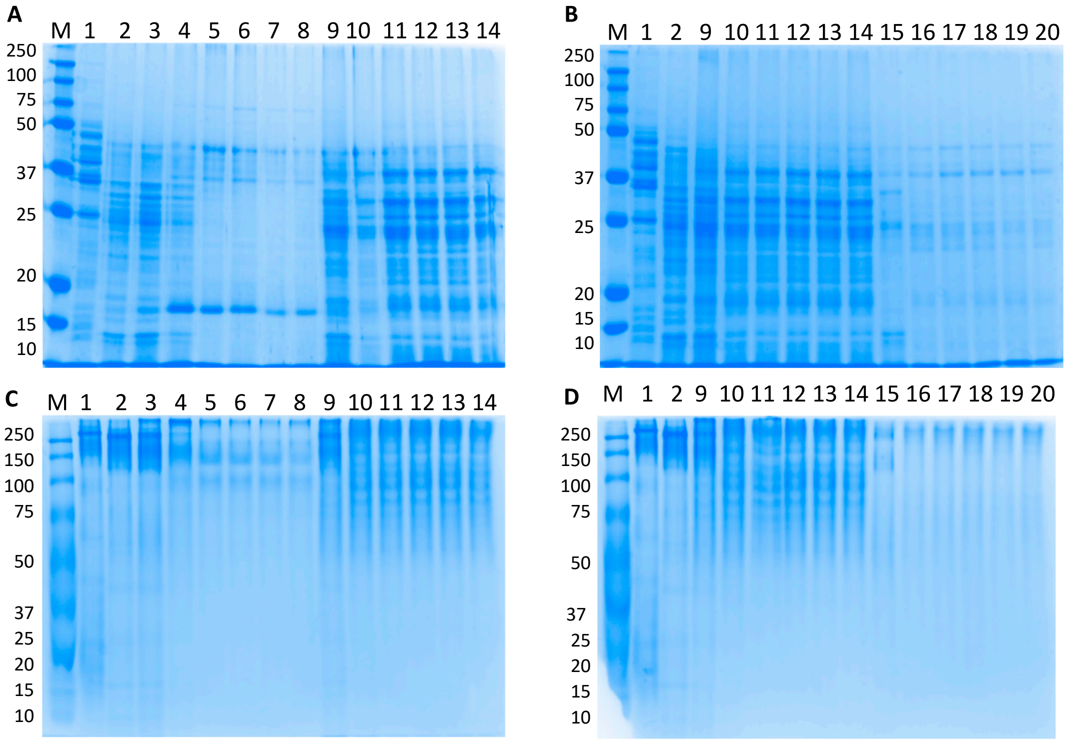

Figure S1. SDS-PAGE run in denaturing (A, B) and PAGE native conditions (C, D) comparing model flour mixtures of lupine in rice and lupine in wheat flours (A, C) and model breads with lupine in wheat flour (B, D). Legend: lane 1, *L. luteus*; lane 2, *L. albus*; lane 3-7, 10%, 1%, 0.1%, 0.01% and 0.001% of *L. albus* in rice flour; lane 8, rice flour; lanes 9-13, 10%, 1%, 0.1%, 0.01% and 0.001% of *L. albus* in wheat flour; lane 14, wheat flour; lanes 15-19, 10%, 1%, 0.1%, 0.01% and 0.001% of *L. albus* in wheat bread; lane 20, wheat bread; lane M, Precision Plus Protein Dual Color Standard 10-250 kDa (Bio-Rad Laboratories, Inc., Hercules, CA, USA).

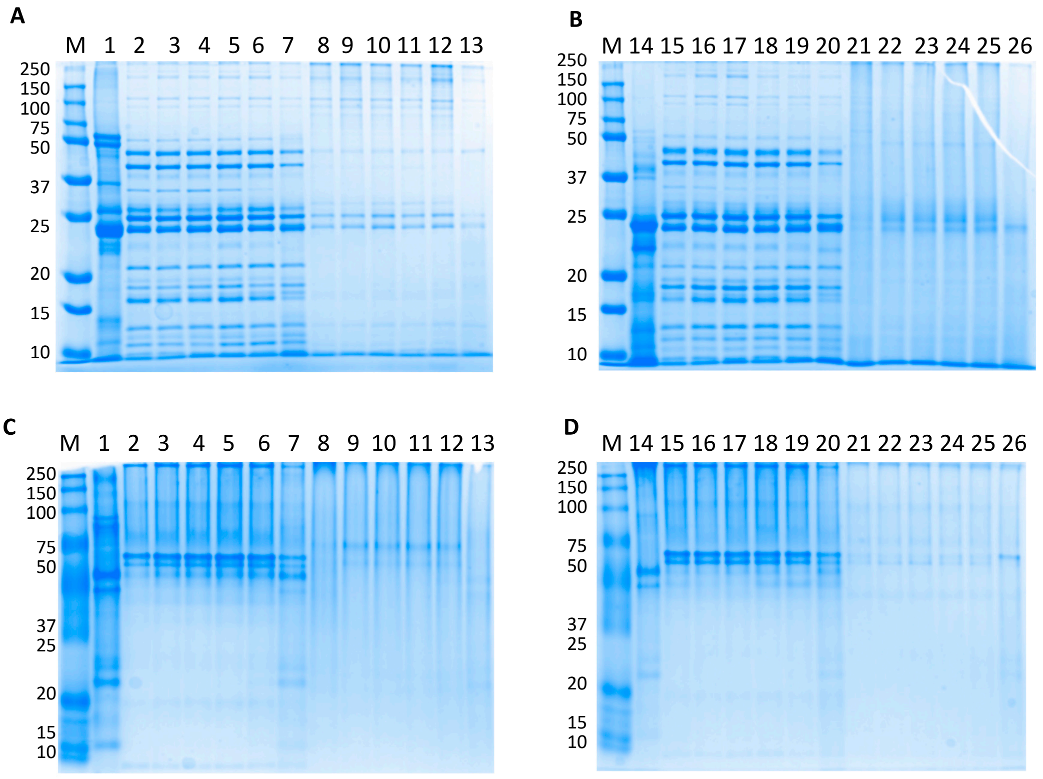

Figure S2. SDS-PAGE gels in denaturing (A and B) and PAGE native (C and D) conditions comparing model mixtures simulating pork hams (A and C) and pork sausages (B and D). Legend: lane 1, SPI; lane 2, raw pork ham; lanes 3-7, 0.001%, 0.01%, 0.1%, 1% and 10% of SPI in raw pork ham; lane 8, cooked-pork ham; lanes 9-13, 0.001%, 0.01%, 0.1%, 1% and 10% of SPI in cooked-pork ham; lane 14, SPC; lane 15, raw pork sausage; lanes 16-19, 0.001%, 0.01%, 0.1%, 1% and 10% of SPC in raw pork sausage; lane 20, autoclaved pork sausage; lanes 21-26, 0.001%, 0.01%, 0.1%, 1% and 10% of SPC in pork sausage (autoclaved); M, Precision Plus Protein Dual Color Standard 10-250 kDa (Bio-Rad Laboratories, Inc., Hercules, CA, USA).

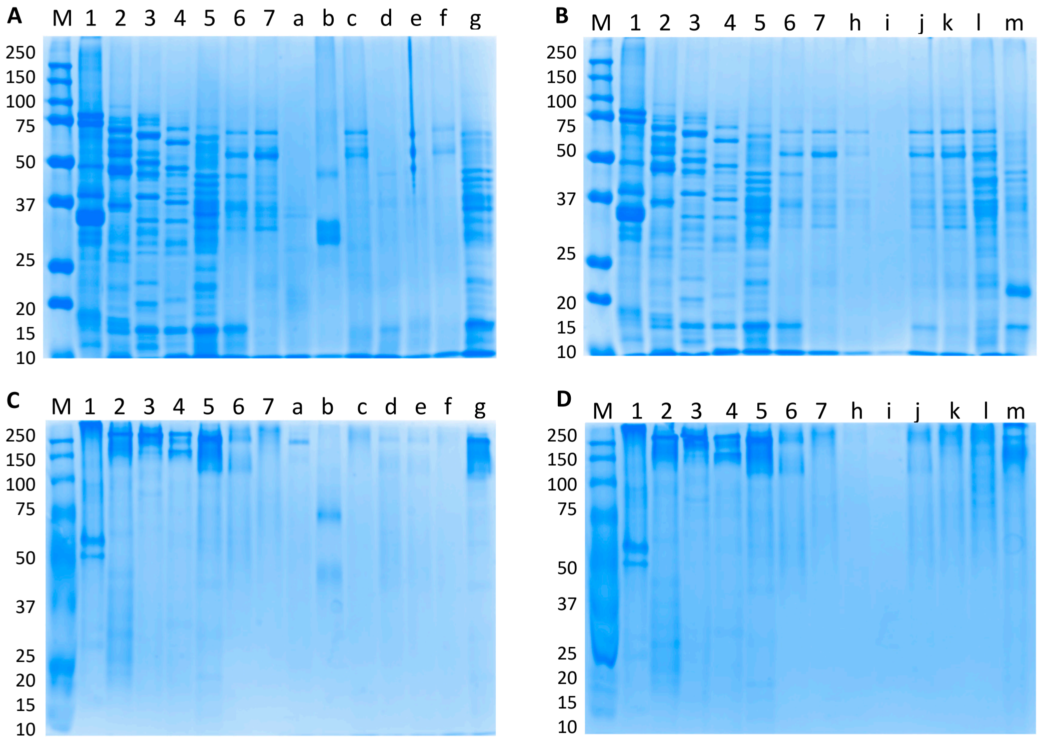

Figure S3. SDS-PAGE run in denaturing (A, B) and PAGE in native conditions (C, D) with commercial samples. Legend: lane 1, SPI; lane 2, *L. luteus*; lane 3*, L. mutabilis*; lane 4, *L. angustifolius*; lane 5, *L. albus*; lane 6, 10% of *L. albus* in bread; lane 7, wheat bread; lane a, cereal bread; lane b, cookies; lane c, lupine biscuits; lane d, Pan Carré; lane e, "crostini"; lane f, lupine cookies with lemon flavour; lane g, flour for bread; lane h, cookies; lane i, chocolate wafers; lane j, model bread containing 2.5% of lupine flour; model bread containing 0.25% of lupine flour; lane l, flour mixture containing 2.5% of lupine in wheat; lane m, flour mixture containing 2.0% of lupine in rice; lane M, Precision Plus Protein Dual Color Standard 10-250 kDa (Bio-Rad Laboratories, Inc., Hercules, CA, USA).

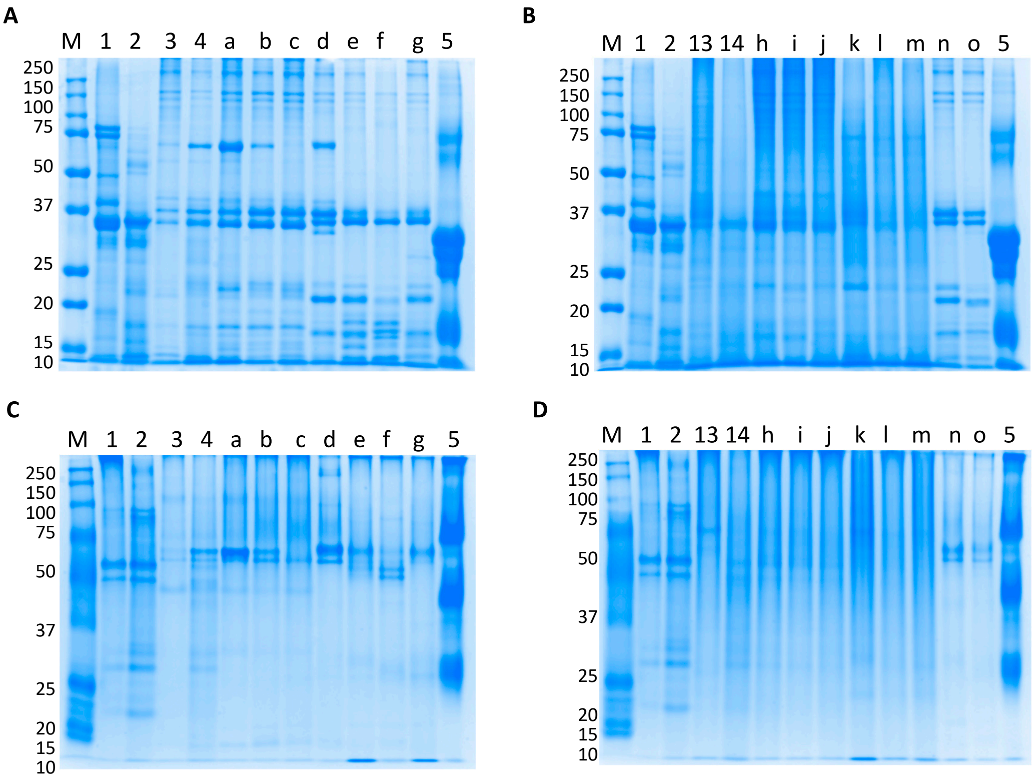

Figure S4. SDS-PAGE gels run in denaturing (A and B) and PAGE native (C and D) conditions with commercial samples of cooked-hams (A and C) and sausages (B and D). Legend: lane 1, SPI; lane 2, SPC; lane 3, cooked-pork ham; lane 4, cooked-pork ham with 10% of SPI; lanes a-c, commercial cooked-pork hams; lane d, cooked-turkey ham; lane e, commercial cooked-turkey ham; lane f, commercial cooked-chicken ham; lane g, commercial cooked-turkey ham; lane 5, milk protein concentrate; lane 13, autoclaved pork sausage; lane 14, 10% of SPC in autoclaved pork sausage; lanes h-j, commercial pork sausages; lane k, autoclaved turkey sausage; lane l, commercial turkey sausages; lane m, commercial chicken sausages; lane n, commercial turkey sausages; lane o, commercial turkey and chicken sausages; lane M, Precision Plus Protein Dual Color Standard 10-250 kDa (Bio-Rad Laboratories, Inc., Hercules, CA, USA),
